# *TERT* Copy Number Alterations, Promoter Mutations and Rearrangements in Adrenocortical Carcinomas

**DOI:** 10.1007/s12022-021-09691-0

**Published:** 2021-09-22

**Authors:** Sounak Gupta, Helen Won, Kalyani Chadalavada, Gouri J. Nanjangud, Ying-Bei Chen, Hikmat A. Al-Ahmadie, Samson W. Fine, Sahussapont J. Sirintrapun, Vivian E. Strong, Nitya Raj, Diane Reidy Lagunes, Chad M. Vanderbilt, Michael F. Berger, Marc Ladanyi, Snjezana Dogan, Satish K. Tickoo, Victor E. Reuter, Anuradha Gopalan

**Affiliations:** 1 Department of Pathology, Memorial Sloan Kettering Cancer Center, 1275 York Avenue, New York, NY 10065 USA; 2 Molecular Cytogenetics Core Facility, Memorial Sloan Kettering Cancer Center, New York, NY USA; 3 Department of Surgery, Gastric and Mixed Tumor Service, Memorial Sloan Kettering Cancer Center, New York, NY USA; 4 Division of Solid Tumor Oncology, Department of Medicine, Memorial Sloan Kettering Cancer Center, New York, NY USA; 5 Present Address: Mayo Clinic, Rochester, MN USA

**Keywords:** *TERT*, Adrenocortical carcinoma, Adrenocortical adenoma, TCGA

## Abstract

Molecular characterization of adrenocortical carcinomas (ACC) by The Cancer Genome Atlas (TCGA) has highlighted a high prevalence of *TERT* alterations, which are associated with disease progression. Herein, 78 ACC were profiled using a combination of next generation sequencing (*n* = 76) and FISH (*n* = 9) to assess for *TERT* alterations. This data was combined with TCGA dataset (*n* = 91). A subset of borderline adrenocortical tumors (*n* = 5) and adrenocortical adenomas (*n* = 7) were also evaluated. The most common alteration involving the *TERT* gene involved gains/amplifications, seen in 22.2% (37/167) of cases. In contrast, “hotspot” promoter mutations (C > T promoter mutation at position -124, 7/167 cases, 4.2%) and promoter rearrangements (2/165, 1.2%) were rare. Recurrent co-alterations included 22q copy number losses seen in 24% (9/38) of cases. Although no significant differences were identified in cases with and without *TERT* alterations pertaining to age at presentation, tumor size, weight, laterality, mitotic index and Ki67 labeling, cases with *TERT* alterations showed worse outcomes. Metastatic behavior was seen in 70% (28/40) of cases with *TERT* alterations compared to 51.2% (65/127, *p* = 0.04) of cases that lacked these alterations. Two (of 5) borderline tumors showed amplifications and no *TERT* alterations were identified in 7 adenomas. In the borderline group, 0 (of 4) patients with available follow up had adverse outcomes. We found that *TERT* alterations in ACC predominantly involve gene amplifications, with a smaller subset harboring “hotspot” promoter mutations and rearrangements, and 70% of *TERT*-altered tumors are associated with metastases. Prospective studies are needed to validate the prognostic impact of these findings.

## Introduction

Programmed loss of telomeric DNA (telomere shortening) in somatic cells leads to the activation of DNA damage responses and subsequent cell cycle arrest and senescence [1]. Telomerase reverse transcriptase, which is encoded by the *TERT* gene, forms an important component of the telomerase complex and TERT mediated de novo telomeric DNA synthesis in rapidly proliferating cancer cells prevents chromosomal ends from being recognized as sites of DNA damage [1]. This prevents the initiation of repair pathways and allows neoplastic cells to escape telomere crisis [1]. Alterations of the *TERT* gene such as “hotspot” promoter mutations, amplifications and promoter rearrangements have been associated with increased *TERT* expression and these alterations have been frequently seen in multiple cancer types [2–6].

Adrenocortical carcinomas are rare endocrine malignancies with an estimated annual incidence of 0.7 to 2 per million and advanced stage neoplasms are associated with extremely poor outcomes [7–12]. Whole genome doubling associated with decreased telomere length and increased *TERT* expression has been associated with disease progression in adrenocortical carcinomas in The Cancer Genome Atlas (TCGA) datasets [12]. A high incidence of genomic amplifications at the *TERT* locus (5p15.33) has also been identified in at least two separate studies (TCGA≈15%, 13 of 89; Assie et al. ≈ 6%, 7 of 122) [12, 13]. In comparison, “hotspot” *TERT* promoter alterations are relatively rare in this tumor type along with *TERT* promoter rearrangements, and the latter are poorly characterized and under-recognized [3, 4, 12, 13]. Due to an association between whole genome doubling and *TERT* expression in a prior TCGA study, it has been suggested that *TERT* is required in a subset of adrenocortical carcinomas for telomere maintenance [12].

As there is a paucity of prognostic biomarkers in adrenocortical carcinomas, we interrogated a large cohort of 169 cases (institutional cohort: 78, TCGA: 91) for *TERT* alterations and correlated the presence of these alterations with various clinicopathologic parameters and outcomes.

## Materials and Methods

### Patient Specimens

**Fig. 1:**
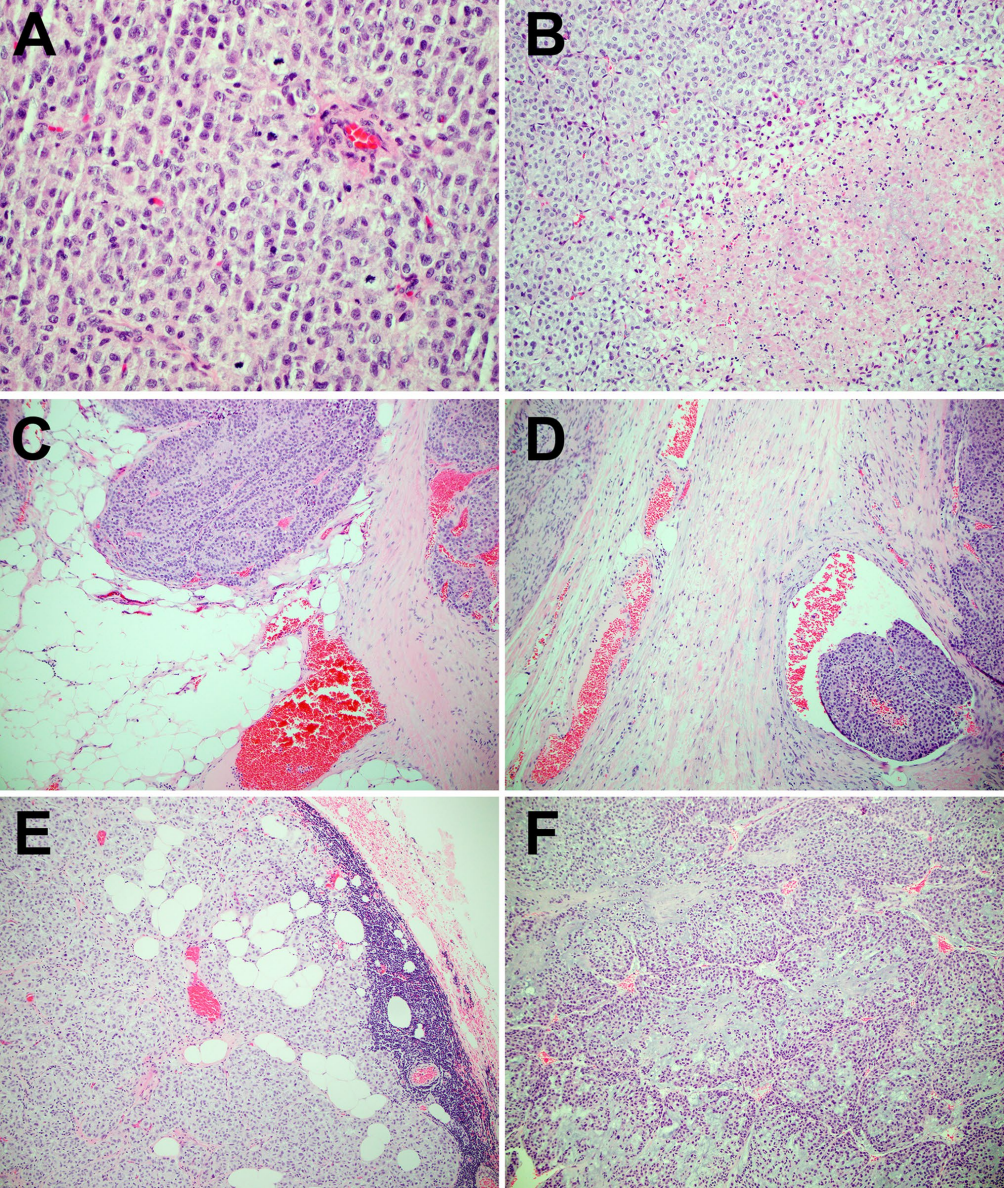
Histopathologic features of adrenocortical carcinomas including high mitotic index (**a**), necrosis (**b**), extra-capsular extension (**c**), lymphovascular invasion (**d**), and lymph node involvement (**e**) have been depicted. An adrenocortical carcinoma with myxoid features (**f**) that harbored a *TERT* amplification has been shown

This study was approved by our Institutional Review Board. Adrenocortical carcinomas were diagnosed using modified Weiss criteria and some of the features of malignant tumors are illustrated in Fig. 1. In addition, the Lin-Weiss-Bisceglia system was used for oncocytic adrenocortical tumors [14–18]. Tumors with a score of 3 under the Weiss system, and those fulfilling 1 to 4 minor criteria under the Lin-Weiss-Bisceglia system were classified as “borderline” in this study. Eighty-seven adrenocortical neoplasia (76 adrenocortical carcinomas, 4 adrenocortical borderline tumors and 7 adrenocortical adenomas) were analyzed by a next generation sequencing (NGS)-based assay, Memorial Sloan Kettering Cancer Center Integrated Mutation Profiling of Actionable Cancer Targets (MSK-IMPACT), as part of an institutional clinical cancer genomics initiative [5, 19, 20]. A subset of 9 adrenocortical carcinomas and 1 borderline adrenocortical tumor were analyzed using fluorescence in situ hybridization (FISH). Cases profiled by FISH included 3 cases that were only profiled using this testing modality (adrenocortical carcinoma: 2; adrenocortical borderline tumor: 1). Archived H&E and immunohistochemical-stained slides were reviewed and medical records were accessed for relevant clinicopathologic features including outcomes on follow up.

### Next Generation Sequencing-Based Molecular Profiling

Specifics regarding the MSK-IMPACT assay have been previously reported [5, 19, 20]. This assay involves hybridization capture-based library preparation followed by deep sequencing of select noncoding regions and 6614 protein-coding exons of 468 genes (previous versions interrogated 341 and 410 genes) including *TERT* [6]. Sequencing 500 bases upstream of the *TERT* promoter allows for the identification of “hotspot” promoter mutations as well as structural variants that may occur in this region [6]. In addition, the homogenous distribution of single nucleotide polymorphism tiling probes across the genome allows for assessment of copy number alterations. Based on previously reported criteria, gains were defined as a fold change ≥ 1.5 and < 2.0, while amplifications were defined as a fold change ≥ 2.0 [21–23].

### Fluorescence *In Situ* Hybridization

Fluorescence in situ hybridization (FISH) analysis was performed on paraffin sections using a 3-color probe set. The probe mix consisted of BAC clones spanning the *TERT* gene (5p15), while probes for the 5p12 and 5q11 loci served as controls. Probe labeling, tissue processing, hybridization, post-hybridization washing, and fluorescence detection were performed according to standard laboratory procedures. Slides were scanned using a Zeiss Axioplan 2i epifluorescence microscope equipped with MetaSystems (Waltham, MA) imaging system. Metafer and VSlide modules within the system were used to generate virtual image of H&E and DAPI-stained sections. For all cases, the corresponding H&E sections assisted in localizing regions of interest for downstream analysis. Signal counts were performed on a minimum of 20 discrete nuclei and only nuclei with at least 2 signals for *TERT* and control probes were selected. *TERT* amplification was defined as ≥ 10 copies of *TERT* or a *TERT*: 5q11 probe ratio > 2. Cells with large clusters of *TERT* signal (high-level HSR type amplification), were interpreted as being amplified.

### Literature Review and Data Extraction from the Cancer Genome Atlas Datasets

The publicly available cBioPortal.32e34 platform was used to analyze data from The Cancer Genome Atlas (TCGA) pertaining to pan-genomic characterization of adrenocortical carcinomas [24, 25].

### Statistical Analysis

Continuous variables were evaluated with frequency counts and percentages and all tests to assess for statistical significance were two-sided and *p*-values < 0.05 were considered significant.

## Results

### Prevalence of TERT Alterations in Adrenal Cortical Carcinomas

76 adrenocortical carcinomas were evaluated for “hotspot” *TERT* promoter mutations as well as for structural variants involving the first 500 bases upstream of the transcriptional start site (Table 1). In addition, using a combination of NGS (*n* = 76) and FISH (*n* = 9), 78 cases of adrenocortical carcinomas were evaluated for copy number alterations (Table 1).

**Table 1:** Prevalence of *TERT* alterations in adrenal cortical tumors

	**MSKCC cohort (ACC)**	**TCGA (ACC)**	**MSKCC and TCGA (ACC)**	**MSKCC (borderline tumors)**
**Total cases**	78	91	169	5
***TERT*** **alterations (%)**	27 of 78 (34.6%)	18 of 91 (19.8%)	45 of 169 (26.6%)	2 of 5 (40%)
***TERT*** **promoter mutations (%)**	3 of 76 (3.9%)	4 of 91 (4.4%)	7 of 167 Cases (4.2%)	0 of 4 (0%)
***TERT*** **amplifications (%)**	24^a^ of 78 (30.7%)	13 of 89 Cases (14.6%)	37^a^ of 167 Cases (22.2%)	2 of 5 (40%)
***TERT*** **copy number (mean fold change by MSK-IMPACT)**	2.1, range 1.5 to 5.8 (*n* = 22)	-	-	1.5 (*n* = 1)
**Structural variants (*TERT*** **promoter)**	1^a^ of 76 (1.3%)	1 of 89 Cases (1.1%)	2^a^ of 165 Cases (1.2%)	0 Cases

No *TERT* alterations were identified for 7 cases of adrenocortical adenomas which were profiled using MSK-IMPACT (including 3 oncocytic adenomas and 1 pigmented adenoma). MSK-IMPACT copy number predictions for *TERT* amplification was confirmed by FISH for 8 cases and copy number assessment was performed using FISH only for 2 cases

^a^A single case had a *TERT* promoter rearrangement in the background of genomic amplification at the same locus

**Fig. 2:**
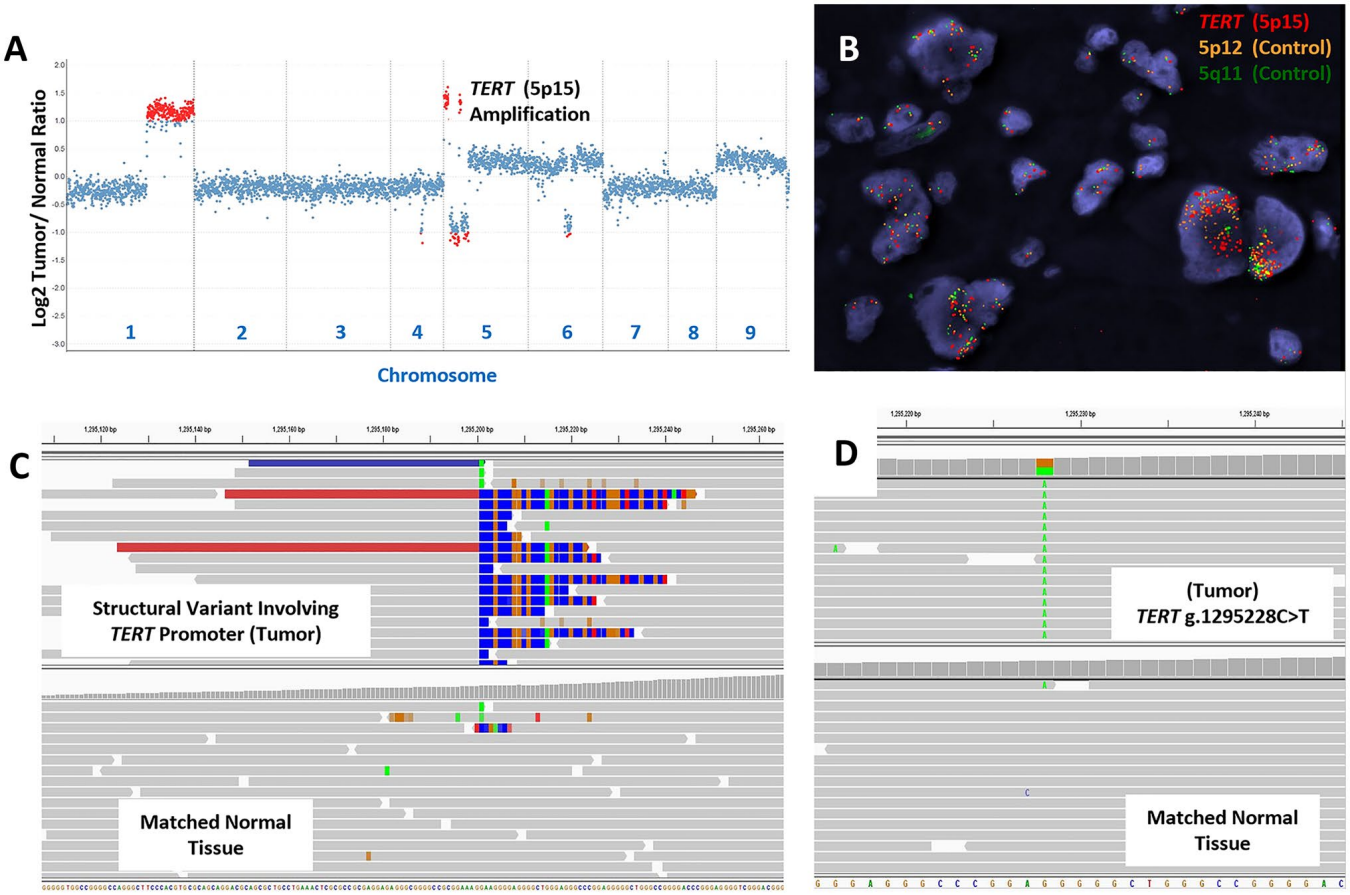
Copy number alterations for an adrenocortical carcinoma, including *TERT* amplification, determined using next generation sequencing and fluorescence in situ hybridization has been depicted (**a, b**). Relative (Log2) tumor/normal ratios (y axis) and corresponding chromosomes (x axis) are displayed, with each blue dot representing an individual probe region and amplified regions are shown in red (**a**). Corresponding fluorescence in situ hybridization results using probes for *TERT* (5p12, red) and control probes (5p12, yellow; 5q11, green) is shown (**b**). A structural rearrangement of the *TERT* promoter region with *CLPTM1L* (**c**), and a somatic *TERT *promoter mutation (g.1295228C > T) (**d**) identified using hybridization-capture based next generation sequencing has been depicted

The most common alteration involving the *TERT* gene involved genomic gains/amplifications which were seen in 30.7% of cases (24/78; Fig. 2A–B; Table 1). After taking into consideration similar events identified in TCGA datasets, the combined incidence for gains/amplifications was 22.2% (37 of 167 cases) [25]. This was significantly higher compared to both *TERT* promoter rearrangements (2/165, 1.2% cases; Fig. 2C; Table 1) and “hotspot” C > T promoter mutations at position -124 relative to the transcription start site (also referred to as the C228T alteration, 7/167, 4.2% cases; Fig. 2D; Table 1) [25]. Interestingly, consistent with a prior TCGA study, no C250T promoter alterations were identified in our institutional dataset [12]. In addition, these *TERT* alterations (gains/amplifications, “hotspot” promoter mutations and promoter rearrangements) were mutually exclusive events.

Of note, 2 of 5 borderline adrenocortical tumors showed similar *TERT* gains/amplifications (Table 1), while no *TERT* alterations were identified for 7 adrenocortical adenomas.

### Clinicopathologic Features of Adrenocortical Carcinomas with *TERT* Alterations

After evaluating abstracted data from institutional cases (*n* = 78) and TCGA cases (*n* = 89), no significant differences were identified regarding mean age at presentation, tumor laterality, size and weight, irrespective of *TERT* alteration status (Table 2). Similarly, while cases with *TERT* alterations tended to have a lower mitotic index and Ki67 labeling (proliferative index), neither of these metrics were statistically significant (Table 3). Interestingly, adrenocortical carcinomas in both the institutional and TCGA cohorts showed a female predilection, irrespective of *TERT* status and the significance of this observation is unclear (Table 2).

**Table 2:** Clinical features of adrenal cortical carcinomas with *TERT* alterations

	**No** ***TERT*** **alteration** **MSKCC cohort (51 of 78 ACC)**	***TERT*** **alteration +** **MSKCC cohort (27 of 78 ACC)**	**No** ***TERT*** **alteration** **TCGA (76 of 89 ACC)**	***TERT*** **alteration +** **TCGA (13 of 89 ACC)**	**No** ***TERT*** **alteration** **MSKCC and TCGA (127 of 167 ACC)**	***TERT*** **alteration +** **MSKCC and TCGA (40 of 167 ACC)**	**No** ***TERT*** **alteration** **(3 of 5 borderline cases)**	***TERT*** **alteration +** **MSKCC (2 of 5 borderline cases)**
**Mean age (years)**	48.6	51.5	46.1	51.4	47.1	51.5	43.3	63
**Sex (Male:Female)**	19:32	11:16	26:49	4:9	45:81	15:25	0:3	0:2
**Laterality (Right:Left)**	14:18	11:16	25:36	7:6	39:54	18:22	1:2	1:1
**Size (cm)**	14.3 (range 4 to 27.2, *n* = 29)	14.3 (range 2.5 to 31, *n* = 20) *p* = 0.2	10.6 (range 2.5 to 20, *n* = 70)	10.1 (7 to 17.1, *n* = 13) *p* = 0.68	11.7 (range 2.5 to 27.2, *n* = 99)	11.2 (range 2.5 to 31, *n* = 33) *p* = 0.68	9 (range 5.8 to 12.5, *n* = 3)	17 (range 15 to 19, *n* = 2)
**Weight (g)**	1019.1 (range 60 to 3790, *n* = 14)	1146.7 (range 77 to 7065, *n* = 10) *p* = 0.96	441.5 (range 22 to 2469, *n* = 62)	300.4 (102.4 to 560, *n* = 11) *p* = 0.35	547.9 (range 22 to 3790, *n* = 76)	655.8 (range 77 to 7065, *n* = 21) *p* = 0.62	230.6 (range 32 to 392, *n* = 3)	1205 (range 1000 to 1410, *n* = 2)
**Clinical follow up (> 3 months)**	27 of 51 cases	21 of 27 cases	40 of 76 cases	8 of 13 cases	67 of 127 cases	29 of 40 cases	3 of 3 cases	1 of 2 cases
**Mean follow up (> 3 months)**	32.4	78.7	56.2	49.5	46.6	74.1	44.3	78
**Documented metastasis at diagnosis/follow up**	36/51 (70.6%)	19/27 (70.4%) *p* = 1.0	29/76 (38.2%)	9/13 (69.2%) *p* = 0.06	65/127 (51.2%)	28/40 (70%) *p* = 0.04	0 of 3 Cases	0 of 1 Case
**Outcome**	AWoD: 3 of 51 AWD: 15 of 51 DOC: 1 DOD: 6 of 51 Unknown: 26 of 51	AWoD: 6 of 22 AWD: 10 of 22 DOD: 6 of 22	AWoD: 5 of 76 AWD: 25 of 76 Unknown: 46 of 76	AWoD: 4 of 13 AWD: 9 of 13	AWoD: 8 of 127 AWD: 40 of 127 DOC: 1 DOD: 6 of 127 Unknown: 72 of 127	AWoD: 10 of 35 AWD: 19 of 35 DOD: 6 of 35	AWoD: 3	AWoD: 1 DOC: 1

*AWoD* alive without disease, *AWD* alive with disease, *DOC* dead of other causes, *DOD* dead of disease

**Table 3:** Histopathologic and genomic features of adrenal cortical carcinomas with *TERT* alterations

	**No** ***TERT*** **alteration MSKCC Cohort (51 of 78 ACC)**	***TERT*** **Alteration + MSKCC cohort (27 of 78 ACC)**	**No** ***TERT*** **Alteration TCGA (76 of 89 ACC)**	***TERT*** **Alteration + TCGA (13 of 89 ACC)**	**No** ***TERT*** **alteration MSKCC and TCGA (127 of 167 ACC)**	***TERT*** **Alteration + MSKCC and TCGA (40 of 167 ACC)**	**No** ***TERT*** **alteration (3 of 5 borderline cases)**	***TERT*** **alteration + MSKCC (2 of 5 borderline cases)**
**Mitosis (per 50hpf)**	39.6 (*n* = 31)	20.1 (*n* = 23) *p* = 0.21	24.7 (*n* = 35)	20 (*n* = 8) *p* = 0.73	31.7 (*n* = 66)	21 (*n* = 31) *p* = 0.26	2 (*n* = 1)	10.5 (*n* = 2)
**Ki67 (%)**	30.3 (*n* = 10)	20.2 (*n* = 13) *p* = 0.26	17.5 (*n* = 30)	17.5 (*n* = 2)	20.5 (*n* = 40)	19.9 (*n* = 15) *p* = 0.69	–	–
**Common associated genomic alterations**	Not evaluated	*TP53* (4 of 25) *CTNNB1* (2 of 25) *NF1* (2 of 25)	Not evaluated	*TP53* (1 of 13) *CTNNB1* (2 of 13)	Not evaluated	*TP53* (5 of 38) *CTNNB1* (4 of 38) *NF1* (2 of 38)	–	–
**Common associated copy number alterations**	Not evaluated	9p21 Loss (*CDKN2A/CDKN2B*): 3 of 25 and 22q Loss (*NF2/SMARCB1*) : 2 of 25	Not evaluated	9p21 Loss (*CDKN2A/CDKN2B*): 3 of 13 and 22q Loss (*NF2/SMARCB1*) : 7 of 13	Not evaluated	9p21 Loss (*CDKN2A/CDKN2B*): 6 of 38 and 22q Loss (*NF2/SMARCB1*) : 9 of 38	–	–

No *TERT* alterations were identified for 7 cases of adrenocortical adenomas which were profiled using MSK-IMPACT (including 3 oncocytic adenomas and 1 pigmented adenoma)

Recurrent alterations that co-occurred with *TERT* alterations included 22q (*NF2/SMARCB1*) copy number losses in 24% of cases (9 of 38). Furthermore, copy number losses at 9p21 (*CDKN2A/CDKN2B*) and alterations involving *TP53*, *CTNNB1* and *NF1* were each seen in ≤ 16% of cases with *TERT* alterations in the institutional and TCGA cases combined (*n* = 38) (Table 3).

For patients with at least 3 months of clinical follow up (institutional cohort, *n* = 48; TCGA, *n* = 48, total, *n* = 96) the presence of *TERT* alterations correlated with adverse clinical outcomes. While TCGA data suggested a trend towards worse outcomes (69.2% vs 38.2%, *p* = 0.06), defined as documented metastatic disease either at diagnosis or on follow up, no such statistically significant differences were identified in the institutional cohort of cases (70.6% vs 70.4%, Table 2). However, at the same time for all cases combined, those with *TERT* alterations (28/40, 70%) had worse outcomes compared to those that lacked alterations of the *TERT* gene (65/127, 51.2%, *p* = 0.04; Table 2), including 6/35 (17.1%) patients with *TERT* alterations dying of disease related complications compared to 6/127 patients that lacked these alterations (4.7%, *p* = 0.02; Table 2). These findings suggest that *TERT* alterations do indeed adversely affect outcomes.

## Discussion

This is a large study involving 181 cases of adrenocortical tumors. A combination of hybrid-capture based next generation sequencing/ fluorescence in situ hybridization (*n* = 78) and whole exome sequencing/targeted *TERT* promoter sequencing (The Cancer Genome Atlas, *n* = 91) was used to define both the prevalence of copy number alterations and “hotspot” promoter mutations affecting the *TERT* gene in adrenocortical carcinomas. Unique findings of this study include understanding the landscape of *TERT* alterations in these tumors which predominantly include gene amplifications, with a smaller subset harboring “hotspot” promoter mutations and rearrangements. The documentation of an increased incidence of metastatic disease (70% vs 51.2%, *p* = 0.04) and disease specific death (6 of 25, 17.1% vs 6 of 127, 4.7%) among patients with known *TERT* alterations in combined institutional/ TCGA cases of adrenocortical carcinomas suggests that these alterations may have significant impact on prognostication. This observation is further underscored by the fact that no *TERT* alterations were identified in 7 cases of adrenocortical adenomas. While *TERT* amplifications were identified in 2 of 5 borderline adrenocortical tumors, clinical follow up was available for only one of these patients and the prognostic significance of this finding in borderline tumors is therefore unclear.

The spectrum of alterations that have been shown to increase *TERT* expression across tumor types are broad [6]. While genomic amplification events for *TERT* at the 5p15.33 locus have been documented in multiple studies of adrenocortical carcinomas, other mechanisms of upregulation of TERT have been documented across various tumor types [6, 12, 13, 26–30]. For instance, G > A substitutions at − 124 and − 146 base pairs upstream of the transcription start site for the *TERT* gene (also referred to as “C228T” and “C250T” promoter mutations) are known to increase recruitment of the heterotetrameric GABP transcription factor complex; this complex binds both at this site and at an adjacent ETS binding site to consequently upregulate *TERT* gene expression [5, 6, 31–33].

Rearrangement events occurring within the *TERT* promoter region have been documented in select tumor types and are thought to upregulate *TERT* expression by juxtaposing the *TERT* coding sequence to strong enhancer elements [3, 4, 6]. While whole genome sequencing approaches have been successful in identifying such alterations, these events are likely under-recognized as most contemporary next generation sequencing assays only sequence a limited portion of the *TERT* promoter [3, 6]. For instance, the MSK-IMPACT assay which sequences the first 500 base pairs upstream of the *TERT* transcription start site was able to identify one such event and in the absence of gene expression data, the functional significance of such alterations remains to be better defined [6]. Only one such case was identified in the cohort of adrenocortical carcinoma profiled by TCGA (TCGA-OR-A5JS) and this case harbored a *CCDC47-TERT* fusion. Due to the rarity of these events in adrenocortical carcinomas there is insufficient data at present to comment on whether *TERT* structural variants in this tumor type exhibit a conserved breakpoint.

In addition, hypermethylation of specific CpG islands upstream of the *TERT* transcriptional start site have been associated with increased *TERT* expression and aggressive disease in both pediatric brain tumors and in adrenocortical carcinomas [30, 34]. However, a recent study suggests that these hypermethylation events are not mutually exclusive with other *TERT* alterations such as promoter mutations and/or copy number alterations [6].

A prior study of pediatric adrenocortical carcinomas had documented a high incidence of *TERT* amplifications in a relatively small cohort of patients (13 of 19, 68.4%) without evaluating for “hotspot” promoter mutation status [26]. In the current study, only a minority of adrenocortical carcinomas occurred in patients less than 20 years of age (institutional cohort: 4 of 78 cases; The Cancer Genome Atlas: 3 of 91 cases) and no *TERT* alterations were documented in these cases.

**Fig. 3:**
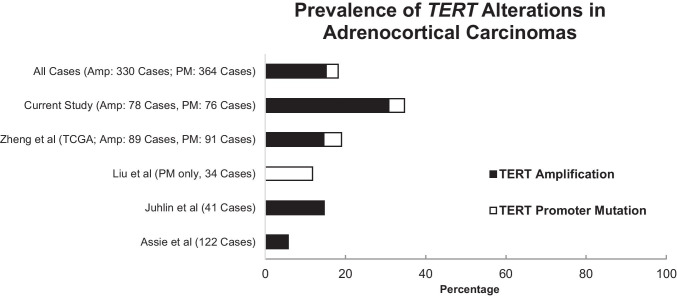
The prevalence of *TERT* alterations (genomic amplifications, black; “hotspot” promoter mutations, white) in the current study, compared to previously reported cases from representative studies in the literature have been shown. The total number of cases that were profiled for amplification events (Amp) and “hotspot” promoter mutations (PM) in each study has been depicted in the figure legend on the left. *TCGA* The Cancer Genome Atlas

After combining the results of our study with *TERT* alterations reported in other studies of adrenocortical carcinomas (Assie et al. 122 cases; Liu et al. 34 cases; Juhlin et al., 41 cases), the overall prevalence of combined amplification (*n* = 330) and “hotspot” promoter mutation events (*n* = 364) was approximately 18.2% (Fig. 3) [13, 27, 28]. Our results suggest that *TERT* gains/amplifications (mean 15.2%, range 5.7% to 30.8%) occur more frequently than “hotspot” promoter mutations (mean 3.0%, range 0 to 11.8%).

With regards to tumors with divergent histologic features, 3 cases of adrenocortical carcinoma with focal myxoid change were identified in a 45-year-old male, 64-year-old female and a 68-year-old female, respectively. All 3 patients had amplifications of the *TERT* gene and had metastatic disease at diagnosis or on follow up, and at least 2 patients were dead of cancer-related causes at last follow up. On histopathologic examination, the mitotic count for these cases was: 2, 5 and 56 per 50 high power fields, necrosis was identified in 2 cases, and capsular and/or vascular invasion was identified in all. None of the cases included in our series was a rare histologic variant. Additional studies are needed to address whether *TERT* alterations are enriched in rarer histologic variants of adrenocortical carcinomas such as the myxoid variant.

Due to the rarity of molecular profiling of adrenocortical carcinomas, data correlating *TERT* status with clinicopathologic variables and outcomes is limited, with at least 1 study suggesting that whole genome doubling associated with increased *TERT* expression has been associated with disease progression in adrenocortical carcinomas [12]. Specifically, abstracted data from institutional and TCGA cases were reviewed to identify potential associations between *TERT* alteration status and clinicopathologic features such as: age at presentation, sex, tumor laterality, size and weight. While no significant trend was identified for these variables in association with *TERT* alterations, a female predilection was identified for combined institutional and TCGA cases (female: 106 of 166 cases, 64%) and supports what has been reported in the literature [8]. Some parameters that have been used for prognostic stratification of adrenocortical carcinomas include: mitotic frequency and Ki67 labeling index [35–40]. However, no significant association between Ki67 labeling index and mitotic activity and *TERT* alteration status was identified in combined institutional / TCGA cases in our study. Finally, recurrent molecular alterations identified in cases with *TERT* alterations included 22q (*NF2/SMARCB1*) and 9p21 (*CDKN2A/CDKN2B*) copy number losses that were identified in 24% and 16% of cases, respectively, and the significance of this finding is unclear, as well.

In contrast to molecular analysis for *TERT* promoter mutation detection which yields a binary result, defining a cutoff for *TERT* amplification (using either FISH or NGS) is somewhat arbitrary. Based on previously reported criteria, gains were defined as a fold change ≥ 1.5 and < 2.0, while amplifications were defined as a fold change ≥ 2.0 for NGS assays. Regarding FISH: ≥ 10 copies of *TERT*, or a *TERT*: 5q11 probe ratio > 2, or large clusters of *TERT* signal (high-level HSR type amplification) met criteria for *TERT* amplification. For 6 cases of adrenocortical carcinomas with *TERT* gene amplifications profiled using both NGS and FISH, the results of both testing modalities were concordant. It must be noted that in contrast to NGS, FISH has the advantages of being a widely available and cost-effective test, with a shorter turnaround time that may be easier to implement into routine clinical practice. However, future studies are needed to further refine both NGS and FISH-based criteria to define amplification in adrenocortical carcinomas.

In summary, the results of our study suggest that *TERT* alterations occur frequently in adrenocortical carcinomas and not in adrenal adenomas, and that copy number gains/amplifications are much more frequent compared to “hotspot” promoter mutations. In addition, *TERT* alterations may be associated with adverse outcomes such as metastatic disease and death from disease. These findings need to be further validated in prospective studies.
